# Probing Molecular Properties at Atomic Length Scale Using Charge-State Control

**DOI:** 10.1021/acs.chemrev.4c00899

**Published:** 2025-06-02

**Authors:** Laerte L. Patera, Shadi Fatayer, Jascha Repp, Leo Gross

**Affiliations:** † Department of Physical Chemistry, 27255 University of Innsbruck, 6020 Innsbruck, Austria; ‡ Applied Physics Program, Division of Physical Science and Engineering (PSE), King Abdullah University of Science and Technology (KAUST), Thuwal 23955-6900, Saudi Arabia; § Department of Physics, University of Regensburg, 93040 Regensburg,Germany; ∥ IBM Research Europe−Zürich, 8803 Rüschlikon, Switzerland

## Abstract

The charge state plays a critical role in governing the structural, electronic, and chemical properties of molecules. Controlling the charge state of individual molecules provides a powerful tool for exploring fundamental processes, such as redox reactions, selective bond rearrangements, molecular excitations, charge transfer, and modulation of reaction pathways at the single-molecule level. Recent advancements in scanning tunneling microscopy (STM) and atomic force microscopy (AFM) have enabled precise and stable manipulation of molecular charge states, allowing for detailed, high-resolution studies of charge-state-dependent phenomena. In this review, we discuss the principles and methodologies for charge-state control in STM and AFM, with a focus on strategies for stabilizing charge states in a controlled experimental environment. We also examine key advancements in the ability to detect and manipulate intra- and intermolecular charge transfer, providing insights into charge-mediated processes, such as structural rearrangements, electronic states, and reactivity at the atomic scale. Finally, we highlight the potential of charge-state control to probe electronic excited states and resolve spin-coherence in individual molecules.

## Graphical abstract

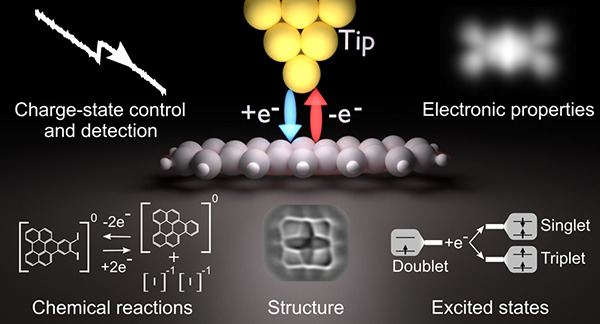

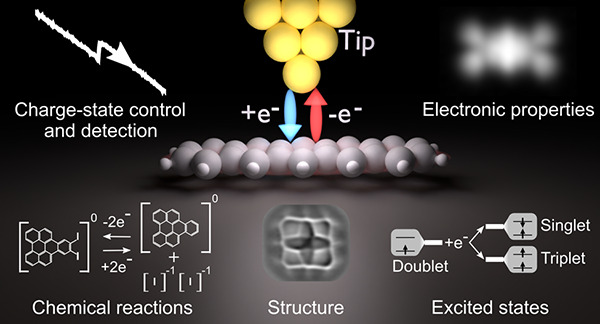

## 1 Introduction

Scanning tunneling microscopy (STM) and atomic force microscopy (AFM) have emerged as powerful tools for characterizing and manipulating individual molecules on surfaces. The combination of atomic-scale imaging, spectroscopy, and atom manipulation has provided unprecedented insights into the atomistic world. Examples of discoveries in the field of scanning-probe-based studies on single molecules range from the direct observation of chemical structures^1^ and relative bond order^2^ to the creation and characterization of elusive molecules,^3,4^ molecular fluorescence,^5,6^ dipolar coupling,^7^ switching between π-diradical open- and closed-shell states,^8^ observation of triplet quenching,^9^ and coherent spin control.^10^

In all these physical and chemical phenomena, the molecular charge state plays a fundamental role, directly affecting key properties such as bond length,^11,12^ molecular conformation,^13,14^ optical behavior,^15,16^ and chemical reactivity.^17,18^ By altering the distribution of electrons, the charge state determines bond order and molecular geometry, influences molecular stability, modulates light absorption and emission properties,^5^ and governs reaction dynamics. Manipulating the molecular charge state provides a versatile strategy for tailoring molecular behavior, enabling control over reaction pathways and facilitating targeted chemical synthesis.^19,20^ In addition, controlled charging in molecular arrays potentially offers a platform for information encoding and processing, in which discrete charge states serve as bits in molecular-scale memory and logic devices.^21^ In catalysis, the molecular charge state plays a pivotal role in determining catalytic activity and selectivity.^22^ For example, in fullerene, C_60_, molecular electrocatalysts, different charge states can lead to substantial variations in catalytic performance.^23^

By controlling the charge state of a molecule and atomically resolving it in different charge states, the influence of excess charges on molecular structural, electronic, and chemical properties can be studied with sub-Angstrom resolution and without ensemble averaging (Figure 1). This approach enables studying redox phenomena at the single molecule level,^24,25^ which we will review here.

**Figure 1:**
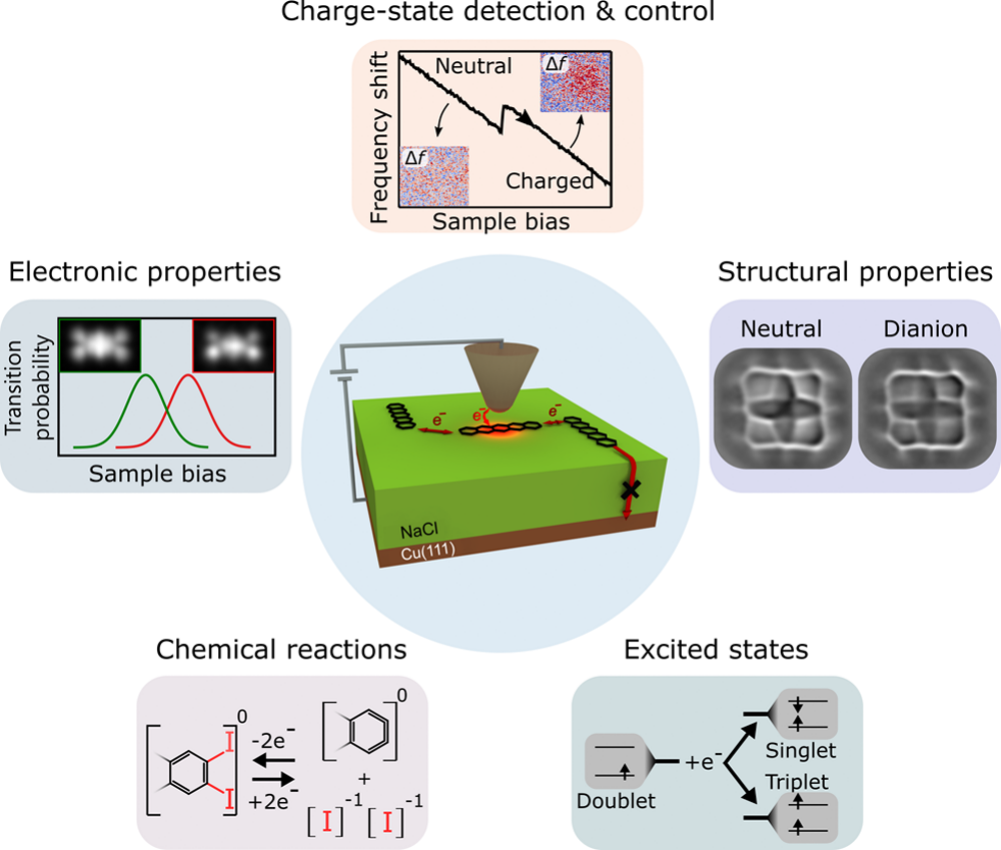
(center) Experimental scheme of probing, controlling, and exploiting charge states of molecules adsorbed on multilayer insulating films using AFM. The film thickness is chosen such that the tunneling of electrons between an adsorbed molecule and the supporting metal is suppressed on experimentally relevant time scales (crossed-out arrow). However, by applying a voltage (sample bias) between tip and supporting metal, electron tunneling between tip and adsorbed molecules can be controlled. Such an experimental setup enables single-molecule charge-state detection and control by AFM. The top panel illustrates how a charging event is detected by AFM, typically observed as a vertical step in the frequency shift (Δ*f*) upon sweeping the sample bias. AFM imaging can reveal the location of the charge. Insets show constant-height AFM data at large tip–sample distances.^26^ By combining charge-state control with the versatile atomic-scale-characterization possibilities of AFM this approach can be used for a detailed examination of the ground-state electronic properties in different charge states (left panel). Insets in the left panel show alternate-charging scanning tunneling microscopy (AC-STM) images, which indicate the impact of excess charges on the spatial distribution of molecular orbitals.^27^ Structural properties in different charge states are revealed by CO-tip AFM data (right panel).^24^ Furthermore, charge-state-induced chemical reactions^28^ and excited electronic states^29^ can be studied using this approach (bottom left and right panel, respectively). Center and top panels adapted with permission from ref 26. Copyright 2015 Springer Nature. Left panel adapted with permission from ref 27. Copyright 2019 Springer Nature. Right panel adapted with permission from ref 24. Copyright 2019 AAAS.

We first introduce the fundamental principles underlying STM and AFM, focusing on their significance in controlling and detecting charges at the atomic scale. The requirements and methods for charge-state control in such experiments are described. Then, we review experiments involving intra- and intermolecular charge-transfer processes as well as the possibilities to induce and probe chemical reactions and molecular structural changes through charge-state control. We also review imaging and spectroscopic approaches for resolving electronic transitions in space and energy. Finally, novel scanning-probe-based methods to access and study electronic excited states via charge-state transitions are discussed.

## 2 Experimental Techniques

### 2.1 Atomic Force Microscopy

Atomic force microscopy (AFM) is a powerful technique that allows the visualization of individual atoms and molecules on surfaces. It is based on the detection of the local force acting between an atomically sharp tip and the sample, and images are obtained by raster scanning the sample surface.^30,31^ Already in the 1990s it has been demonstrated that AFM is sensitive enough to probe individual elementary charges.^32^ For high-resolution imaging, AFM is usually operated in the frequency-modulation noncontact mode (NC-AFM), in which the cantilever holding the tip is oscillated with constant amplitude at its resonance frequency.^33^ The tip–sample interactions cause a resonance-frequency shift, Δ*f*, representing the primary experimental signal in NC-AFM. For small oscillation amplitudes *A* (on the order of 1 Å), Δ*f* is proportional to the vertical derivative of the vertical force component acting between tip and sample.^34,35^ The relative sensitivity to short- and long-range forces depends on *A*.^31^ Amplitudes on the order of one Å peak-to-peak^31^ are typically employed for obtaining high-resolution, atom- and bond-resolved images.^1^ The relatively stiff qPlus force sensors^36,37^ are very well suited as they allow stable operation at such small amplitudes and are typically employed in the reviewed experiments. For detecting electric charges (see below), often slightly larger amplitudes, on the order of a few Å, are employed.^38^

Besides Δ*f* there is a second experimental signal in NC-AFM, the so-called dissipation signal, in most cases reflecting the mechanical damping of the cantilever oscillation. If the force experienced by the tip differs during inward and outward motion of the cantilever hysteretically, it will cause additional damping or excitation, which will be detected in the dissipation signal.

Achieving high-resolution imaging with AFM crucially depends on the characteristics of the tip’s terminal atoms,^1^ which can be controlled by picking up individual atoms or molecules from the surface. The molecular structure of individual molecules on surfaces can be resolved by AFM using functionalized tips.^1,39^ The imaging mechanism for atomic resolution on molecules relies on the chemical inertness of the tip to access the Pauli-repulsion regime, whereas van der Waals and electrostatic forces add a background with little corrugation on the atomic scale. Most prominently, CO tip-functionalization^1^ achieves atomic, bond-resolved contrast with AFM. The lateral deflections of the CO molecule enhance contrast and provide apparent image sharpening, revealing detailed insights into molecular structures and facilitate bond-order analysis.^2^ Several alternative tip functionalizations, such as Xe, Kr, NO, Br,^40^ Cl,^1^ N_2_O,^41^ CuOx,^42^ and larger aromatic molecules,^1^ offer specific contrast differences and can be advantageous depending on the aim of the study. From a technical point of view, bond-resolved AFM imaging with functionalized tips usually requires ultrahigh-vacuum conditions (UHV), as well as cryogenic temperatures, to ensure surface cleanliness, mechanical stability, and immobilization of molecules on the substrate.

### 2.2 Kelvin Probe Force Spectroscopy

Kelvin probe force microscopy (KPFM) and Kelvin probe force spectroscopy (KPFS) are AFM-derived techniques that allow resolving local electric charges and differences of the electric potential on the sample surface.^43^ These methods trace back to Lord Kelvin’s proposition in 1898, which involved determining the work function of a conductor relative to a known reference material. When two metals with different work functions, Φ_1_ and Φ_2_, are electrically connected, their Fermi levels (*E*_F_) align, resulting in a potential drop **V*_CPD_* = (Φ_1_ – Φ_2_)/*e* across the vacuum barrier, see Figure 2a. In AFM, the two metals are the tip and the sample, respectively, and the electric field associated with the potential drop in the tip–sample junction leads to a measurable electrostatic attraction between tip and sample. By applying a voltage compensating the electrostatic attraction, the contact potential *V*_CPD_ can be determined. Specifically, by measuring Δ*f* versus sample bias (*V*) typically a parabola is obtained, in which the voltage V* corresponding to the Δ*f*-maximum of the parabola is referred to as the local contact potential difference (LCPD).^44,45^ Because the tip–sample junction has a different geometry than that of a plate capacitor, the electric field is inhomogeneous. Consequently, at *V* = V* = *V*_LCPD_ the field is not nullified but only minimized. With decreasing tip–sample distance the LCPD becomes increasingly sensitive to the specific sample location and can reach atomic-scale resolution.^46–48^ With KPFS the charge state of individual atoms can be determined, as shown in Figure 2b–d,^48^ for individual gold adatoms that are stable in two different charge states.^49^ Depending on whether the gold atom is charged or neutral, the local electric field differs, resulting in different V*. In general, the direction of the change of the LCPD, that is, of V*, indicates the direction of the change in the charge state: A more negative charging of the sample (adsorbate) results in a shift to larger, i.e., more positive LCPD. Thus, measuring the LCPD on the atomic scale enables the differentiation between positively charged, neutral, and negatively charged adatoms and thus detecting an elementary charge on individual atomic sized adsorbates by means of AFM.^48^ Expanding further on this, the specific value of the measured LCPD depends on the atomic structure of the tip apex, and on the tip-to-sample distance, because of averaging effects.^47^ Therefore, ideally, KPFM measurements are performed with identical tip and for identical geometries and identical tip–sample distances. For such comparative measurements, the direction of the LCPD shift is expected to correspond to the direction of the change of the charge state, that is, a shift to larger (smaller) LCPD for a more negative (positive) charge state of the adsorbate.^47,48^ To account for possible charge-induced changes of the adsorption height, affecting the molecule-to-sample distance, it is beneficial to compare measurements for a variety of tip heights, in order to estimate the effect of the different geometries.^47^

**Figure 2:**
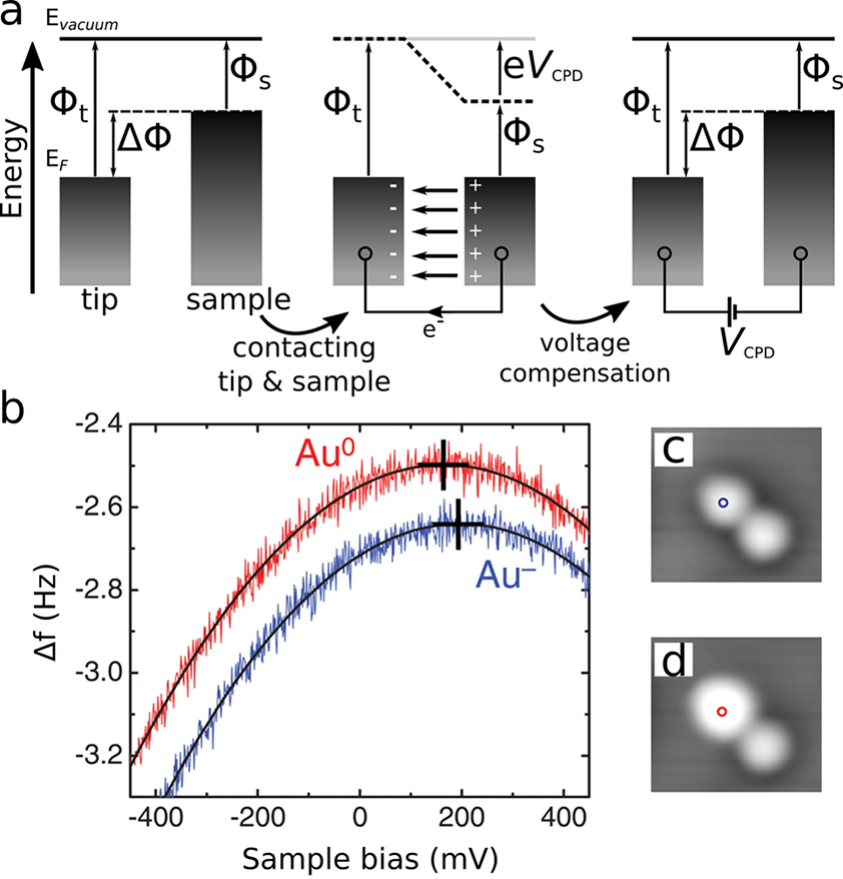
(a) Working principle of Kelvin-probe methods, depicting the energy levels of tip and sample having work functions Φ_t_ and Φ_s,_ respectively, differing by ΔΦ. Upon electrically connecting the metals, electrons flow until their Fermi levels (*E*_F_) are aligned, resulting in an electric field in the junction. The surface charges creating the field lead to an attractive electrostatic force between the electrodes. By applying a voltage *V* = *V*_CPD_, the contact potential difference is compensated for and the electric field is nullified (assuming an ideal plate capacitor geometry). (b) Measured Δ*f*(*V*) above an individual anionic and neutral gold adatom.^48^ The voltage of the peak of the so-called Kelvin parabola is the local contact potential difference (LCPD), minimizing the electric field in the junction. (c) and (d) STM images taken before and after applying a bias pulse and manipulating the charge state of a gold adatom on bilayer NaCl on Cu(111) from anionic to neutral, respectively. (b–d) Adapted with permission from ref 48. Copyright 2009 AAAS.

### 2.3 Scanning Tunneling Microscopy

First evidence of atomic-scale charge detection, bistability and manipulation had been obtained by scanning tunneling microscopy (STM), for atoms and molecules deposited on ultrathin insulating films.^49–51^ In the following, we describe STM in this particular context.

STM is a powerful imaging technique^52^ that provides access to individual atoms and molecules on surfaces. In contrast to AFM, it works by measuring the tunneling current between the tip and a conductive sample surface, while applying a bias voltage across the tip–sample junction.^53^ For tip–sample distances on the Angstrom scale, electrons tunnel through the vacuum gap, creating a measurable current. This current strongly depends on the tunneling distance and can therefore be used to infer the surface topography. However, the sample’s electronic properties also affect the tunneling current. In scanning tunneling spectroscopy (STS), the differential conductance (d*I*/d*V*) is directly related to the local density of states of the sample. A combination of STM and STS allows the characterization of the electronic structure and frontier orbital densities of individual molecules.^54^ However, when molecules are adsorbed on a metallic surface, their electronic coupling with the substrate leads to hybridization, profoundly altering the molecule’s properties. To prevent this, ultrathin insulating layers,^54–56^ typically few monolayers (ML) thick, have been introduced. Common choices are alkali halides (NaCl, RbI, KBr), as well as oxides (alumina and MgO) layers grown on top of atomically flat metal surfaces.^51,57,58^ Such ultrathin insulating films on a metallic substrate effectively suppress hybridization of the adsorbate’s electronic states with the ones of the substrate and thereby preserve (to some extent) the electronic states of individual molecules for examination. At the same time, ultrathin insulating layers, being only few atomic layer thick, still facilitate electron tunneling, leading to a finite junction conductance as required for STM imaging.^55,57^

## 3 Charge-State Bistability on Ultrathin Insulating Films

The controlled manipulation of atomic charge states has been reported in 2005 for individual gold adatoms deposited on ultrathin insulating sodium chloride film using STM.^49^ The charge state of the Au adatoms could be reversibly switched between the neutral and anionic states by applying a sample-bias pulse with the STM tip. Importantly, the charge-state bistability is not only due to the insulating property of the underlying film. It results because large ionic relaxations occurring within the polar NaCl film strongly stabilize the excess charge by a large reorganization energy. For charge-state bistability of adsorbates on ultrathin insulating films the work function of the metal support is important, as it affects the relative energies of neutral and charged states and can be used to tune charge-state bistability.^59^ Exploiting the polaronic relaxation of the ultrathin dielectric support, charge-state bistability has been reported also in other systems,^59–62^ and could be used to reveal distinct changes in the molecular conformation upon charging.^63^ Importantly, STM mapping of several molecular resonances can be used to assign charge states on ultrathin insulating films.^62^

Overall, STM experiments on ultrathin insulating films (typically 1–3 ML) demonstrated how to combine charge-state control with the STM-specific possibilities of probing the distribution of electronic states in space and energy.^54^ However, the charge-state bistability on ultrathin insulating films relies on a delicate energy balance, which is met only for a limited number of molecule/substrate combinations, but not in general, restricting the choice of molecular systems that exhibit charge-state bistability on ultrathin insulating films.^25,59–65^

## 4 Nonconducting Films to Stabilize Charge States

On thicker insulating films,^66^ tunneling of electrons between adsorbates and the metal substrate can be strongly suppressed. For NaCl, every additional atomic layer reduces the tunneling rate through the film by roughly 1 order of magnitude,^66^ such that already films of a moderate thicknesses of above 14 ML^66,67^ suppress electron tunnelling to or from the underlying metal substrate on relevant time scales. Henceforth we refer to these films as thick films, although in other scientific contexts 20 ML might be considered thin. The use of such thick films precludes the use of STM, because no direct current flows between the tip and substrate. Therefore, studies on thick films rely on AFM detection, and charges are only injected by tunneling from/to the tip but not from/to the sample. To change an adsorbate’s charge state on such thick insulating films, the AFM tip can be used to inject charges locally^26^ (see Figure 3a). For thick insulating films, the voltage between the tip and metal support gates the molecular electronic levels with respect to the chemical potential (Fermi level) of the conductive AFM tip. Hence, the sample bias has now the role of a gate voltage, controlling charge exchange between tip and molecule (see Figure 3b).

**Figure 3:**
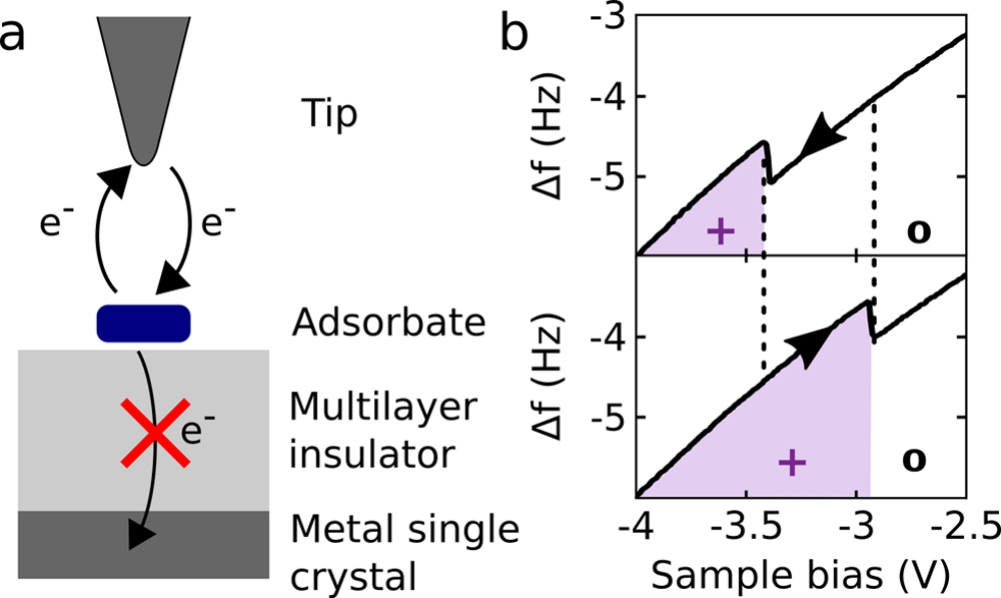
(a) Schematic of an AFM experiment enabling charge-state control. The transfer of electrons can occur only between tip and adsorbate but not between adsorbate and metal substrate for energies within the bandgap of the insulator. (b) KPFS, i.e., Δ*f*(*V*) spectra, showing the manipulation of charge state of a molecular adsorbate. Sweeping the bias downward (upward) an electron is detached from (attached to) the adsorbate, see upper (lower) panel. The hysteresis between electron attachment and detachment is related to the reorganization energy (Figure 8), giving rise to a voltage region of charge-state bistability. Adapted with permission from ref 26. Copyright 2015 Springer Nature.

The charge state of an adsorbate on a thick NaCl film can be controlled by the sample bias *V* when the tip is in tunneling distance. In Figure 3b Δ*f*(*V*) spectra are shown for different sweep directions of *V*. Vertical steps in the spectra indicate the switching of the charge state of the adsorbate. A step upon sweeping the voltage in the positive (negative) direction indicates the addition (removal) of an electron from the tip to the adsorbate. In the central region of the graphs, a hysteresis is visible. That is, the voltage of charge-state switching (here between cation and neutral adsorbate) depends on the direction of the sweep, and thus on the history of the experiment. The hysteresis is related to the reorganization energy, which can be extracted; see below. Importantly, as the switching is a single tunneling event, the specific voltage of the charge-state switching for a given event has also a stochastic component,^38^ and the voltage of charge-state switching also depends on the tip height (affecting the tunneling probability) and the voltage-sweep rate. The voltage region of charge-state hysteresis, and thus charge-state bistability, is important and utilized for many experiments reviewed here, e.g. determination of reorganization energy (section 8.1),^38^ alternate-charging scanning tunneling microscopy (AC-STM, section 8.3),^27^ excited-state spectroscopy (section 9),^29^ triplet lifetime measurements (section 9),^9^ and electron spin resonance atomic force microscopy (ESR-AFM, section 10).^68^

Voltages outside the regions of hysteresis can be used to set the molecules reliably to the desired charge states. For the example shown in Figure 3b, with the tip in tunneling distance, the molecule will always become neutral for *V* = −2.5 V and always cationic for *V* = −4.0 V. At such voltages applied, the molecule can be investigated in the respective charge states (see section 7). If the tip is retracted at such a sample bias, the adsorbate will keep that charge state. As tunneling to the tip is needed to change the charge state, the set charge state will remain while the tip is not in tunneling distance, even if the sample bias is changed after the tip has been withdrawn from the molecule. In this way, different charge states can be set with the tip in tunneling distance first, and then the resulting charge states can be measured with AFM at a tip distance far enough to avoid changing the charge states by tunneling to the tip (see section 5).

Assigning the charge states requires one of the accessible charge states, corresponding to a section of a KPFM parabola without a step, to be known. Starting from that assigned charge state, all other charge states can be assigned assuming that single electrons are added (removed) at steps in the KPFS parabolas, when the bias voltage is swept upward (downward). In most systems the charge state around zero bias is the neutral charge state.^24^ However, for adsorbates with a large value of electron affinity (especially on samples with a small work function) the adsorbate might be charged negatively at *V* = 0 V, as for example for the case of iodine adatoms on NaCl on Cu(111).^28^ Likewise, a small ionization energy (especially in combination with large sample work function) might lead to the cationic state at *V* = 0 V. Note that the charging voltages are affected by the work functions of the substrate system.^59^ The charge state at zero bias can be experimentally determined by investigating the same system but with a bilayer film thickness and from there extrapolating to the thick film. For thin films on coinage metal (111) surfaces the charge state at *V* = 0 V (or if there is a charge-state transition near *V* = 0 V, the two corresponding bistable charge states) can be determined from STS and observation of interface-state scattering: Neutral adsorbates barely scatter interface-state electrons, whereas charged adsorbates do. Anionic adsorbates do not show interface-state localization, but cationic species do.^49,59^

The number of accessible charge states is limited, because for energies outside the electronic bandgap of the insulating support, the insulating film allows electron conduction.

As mentioned, the occurrence of an electron transfer between tip and an adsorbate can be probed by AFM as a sudden change of the resonance frequency Δ*f*, in response to the different electrostatic force acting on the tip (Figure 3b).^26^ The step in the Δ*f* signal is related to the transition between the different Kelvin parabolas. Therefore, when a charge-state transition occurs at voltages close to the crossing points of the respective parabolas, the charging fingerprint will be challenging to detect in the Δ*f* signal.

On thick films, the application of a sample bias does not result in a steady-state current through the molecule. Therefore, on thick films, AFM images can be taken at larger sample bias (within the bandgap of NaCl), in contrast to the situation on thin films. This way, the charge-state control described above can be directly combined with AFM imaging in multiple charge states, including bond-order analysis (see section 7).^24^

To quantitatively extract energy levels (see section 8) from the sample bias, at which transitions are observed, the voltage drop across the dielectric film should be considered. A simple model relies on two planar capacitors connected in series, of which one capacitor corresponds to the vacuum region between the tip and the surface of the NaCl film (*C*_vac_), whereas the second capacitor corresponds to the dielectric film region (*C*_dielectric_), having a dielectric constant (*ε*) and thickness (*d*). Assuming a plate capacitor geometry, the voltage across the dielectric region *V*_dielectric_, when applying *V* as sample bias, can be expressed as

1 $$V_{\mathrm{dielectric}}=\frac{V}{1+\frac{\varepsilon_{\mathrm{dielectric}}d_{\mathrm{vac}}}{\varepsilon_{\mathrm{vac}}d_{\mathrm{dielectric}}}}$$

A more realistic approach involves simulating the voltage drop for a 3D geometry of the tip.^38^ In this context, a finite element method allows the incorporation of the essential geometrical and electric constraints needed to numerically solve the Laplace equation for the electrostatic potential. Such approach yields a relative voltage drop of about 17% through a 14 ML NaCl for a tip-molecule distance of 20 Å.^38^ For comparison, the 1D plate capacitor model yields a voltage drop of 25% for the same thickness of the vacuum and dielectric film.

As a result of the voltage drop through the dielectric (*V*_dielectric_, see eq 1), the effective shift Δ*E* of the energy levels induced at the adsorbate upon applying a sample bias *V* is given by

2 ΔE=eαV

where *e* is the elementary charge and α is the lever arm, a proportionality factor quantifying the fraction of the applied voltage, which drops between tip and adsorbate, giving rise to a shift of the adsorbate’s energy levels.^69^ The effect of the lever arm was measured on NaCl films,^57,70^ and was also observed for molecules adsorbed on metals, indicating a partial drop of the voltage between adsorbed molecule and metal surface.^69,71,72^ In addition to the applied bias, contact potential differences between tip and sample contribute to tip-height dependent shifts of the adsorbate’s energy levels.^71^

Another approach to relate the sample bias to a quantitative energy scale is to gauge the former against an energy difference that is known from other experiments. In particular, the excitation energy of the first excited singlet state is often known from luminescence and can be used to gauge the lever arm of the sample bias acting as a gate. Such a gauge is entirely based on experimental observations, and by coadsorbing a well-studied molecular species, this gauge can also be applied to unknown compounds.^73^

## 5 Intermolecular and Intramolecular Single-Electron Transfer

The capability to control and detect the charge states of individual molecules on thick insulating films allowed the study of inter- and intramolecular charge-transfer processes.

The controlled transfer of single electrons between two close-lying pentacene molecules has been reported (see Figure 4a).^26^ In this experiment, the AFM tip was used to control electron tunneling between the tip and molecules as explained above, but at the same time, the inhomogeneous electric field in the AFM junction gated the transport of an electron between two molecules on the thick NaCl film. Specifically, above two initially neutral molecules (configuration “00” in Figure 4a,b), a negative sample bias was applied to tunnel one electron from the molecule under the tip to the tip, leading to configuration “+0”. At this gating condition, the tip’s potential is attractive for electrons and acts stronger on the molecule directly beneath the tip as compared to the neighboring pentacene molecule. When the sample bias was further reduced to a more negative value, an electron tunneled from the neighboring molecule to the molecule under the tip, leading to configuration “0+”. Further decreasing the sample bias leads–once again–to the tunneling of an electron of the neutralized molecule under the tip into the tip, leaving two positively charged molecules, configuration “++”. Each of these three charge-transfer processes is seen as a sudden step in the Δ*f*(*V*) signal (see Figure 4a), and the aforementioned assignment to the individual processes is confirmed by AFM images, at an increased distance at which the charge states are not affected, after each single electron transfer (see Figure 4b).^74^

**Figure 4:**
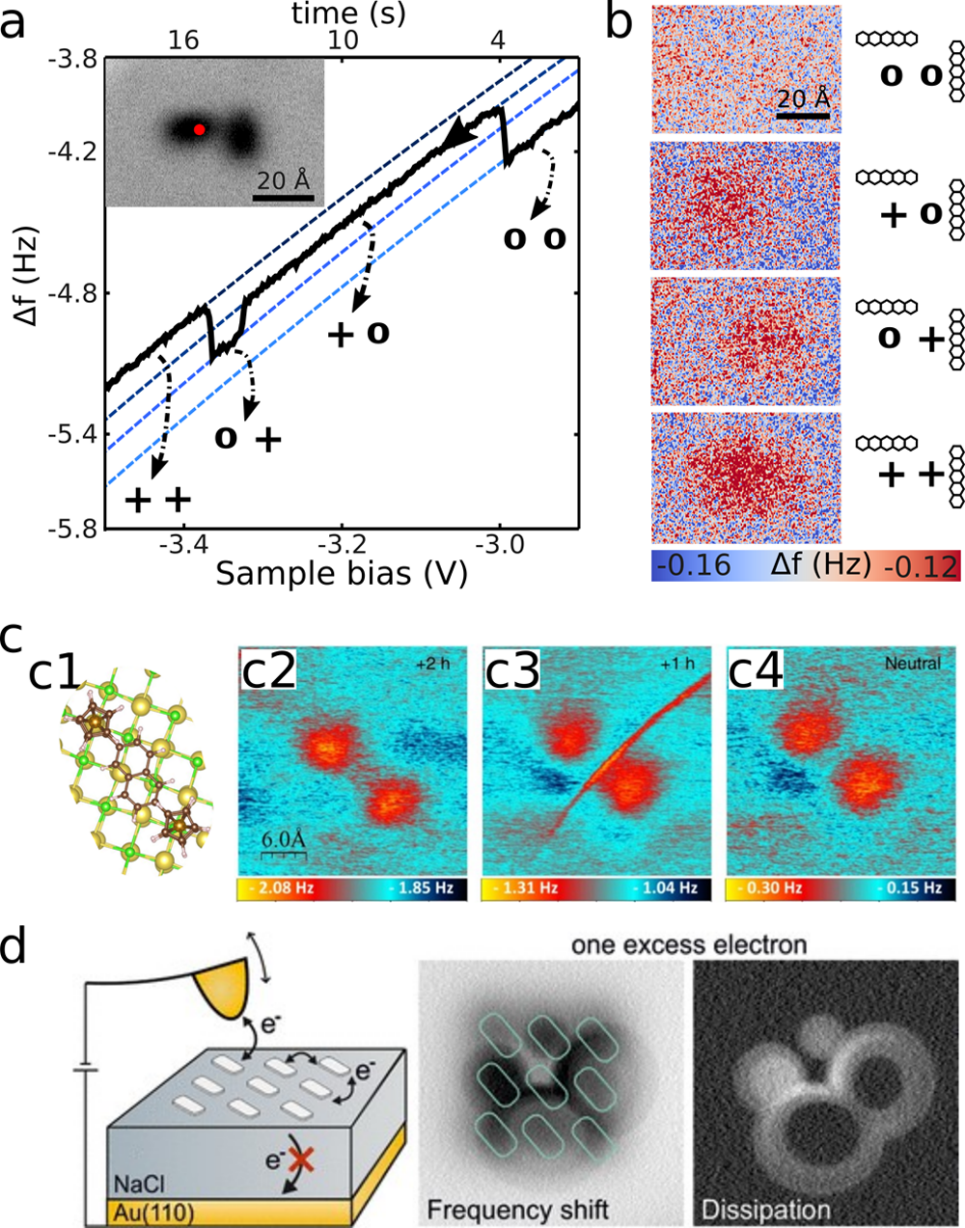
(a,b) Lateral charge transfer between individual molecules.^26^ (a) Δ*f*(*V*) spectrum performed on top of one of the pentacene molecules shown in the inset (constant-height AFM image). Each step in Δ*f* indicates a single charge-transfer process, with individual segments of the Δ*f*(*V*) spectrum corresponding to different charge configurations. (b) Constant-height AFM images revealing the locations of excess charges. A representation of the corresponding charge configurations is shown next to each image. (c) Structural model (c1) and constant-height Δ*f* images (c2–c4) of a molecule featuring two redox centers separated by a linker; (c2) dication, that is, after attachment of two positive charges, +2h (two added holes); (c3) cation, after attachment of one positive charge, +1h (one added hole); (c4) neutral charge state.^74^ The sharp line observed in the Δ*f* image in the cationic state reflects the shuttling of charge between two redox centers of the molecule induced by the tip oscillation. (d) Schematic illustration (left) of the AFM measurement of a molecular assembly. Constant-height AFM images of an assembly of three-by-three molecules (positions as indicated in turquoise) showing the Δ*f* (center) and the dissipation (right) signals.^83^ (a,b) Adapted with permission from ref 26. Copyright 2015 Springer Nature. (c) Adapted with permission from ref 74. Copyright 2020 Springer Nature. (d) Reproduced with permission from ref 83. Copyright 2020 American Chemical Society.

Because of the oscillation of the AFM tip, the gating effect of excess charges by the tip is also oscillatory since it depends on the molecule-tip distance *d*_vac_ (see eq 1) – in other words, the lever arm α oscillates in time, synchronized with the AFM tip. If an energy level of the adsorbate is shifted close to the Fermi level of the tip (or of the back electrode, or to a different energy level of an adsorbate, depending on the measurement scheme, see below) by gating from a constant sample bias (see eq 2), the oscillatory gating given by the tip can lead to alternating electron transfer that is then synchronized with the cantilever oscillation.^69,71,75,76^ The AFM detection is particularly sensitive to oscillatory forces synchronized with the cantilever’s motion. Therefore, an alternating electron transfer can be readily detected in the Δ*f* signal and often also in the dissipation signal^75,77^ (see section 2.1). Whether a periodic charging is seen in the Δ*f* or in the dissipation signal depends on the relative timing between cantilever oscillation and charge transfer and can therefore provide information about tunneling rates.^78,79^ The lateral distance between the tip and the localized state associated with the charge transfer also matters (not included in eq 1), and only at a certain lateral distance does the gating from the tip lead to charge transfer. Therefore, such a gating effect is often observed as a ring-like feature around the localized state, examples of which are discussed further below. The ring-like features associated with charging are also observed in STM/STS experiments.^80–82^ In AFM they can be observed in the Δ*f* and/or the dissipation signal, depending on the details (see below).^74,77,78^

Intramolecular charge transfer has been detected in the Δ*f* and the dissipation channel within a single AFM experiment.^74^ To this end, a molecule consisting of two redox centers separated by a linker was investigated. The charge state of the molecule could be controlled to be neutral and singly and doubly positive by the sample bias, as explained above. When charging the molecule positively by one elementary charge, a sharp line appeared in Δ*f* AFM images, separating the two redox centers (Figure 4c, panel c3). This line was absent in the case of a neutral and dicationic molecule (Figure 4c, panels c4 and c2, respectively). This agrees with the interpretation that for the singly cationic state, the position of the tip will determine in which of the two redox centers the excess charge is located. On the sharp contrast, the excess charge is alternatingly shuttled back and forth between the redox centers. Such a sharp feature was also present in the dissipation signal,^74^ lending further support to this interpretation.

A similar detection mechanism was used to observe intermolecular electron hopping in ordered nanometer-sized islands resulting from the self-assembly of molecules on NaCl.^83^ In an island consisting of a three-by-three arrangement of nine molecules, up to four excess electrons were injected in a controlled manner. Depending on the number of excess electrons, different patterns in the Δ*f* AFM images and the dissipation signal occurred (Figure 4d). This data could be interpreted in terms of local gating by the presence of the AFM tip^84^ and the alternating transfer of electrons between molecules within the island. In a different work, molecular islands on top of a calcite substrate could be charged with single electrons by the AFM tip at room temperature.^85^ These studies show how AFM can shed light on the distribution and transfer of strongly localized charges and their mutual interactions in molecular arrangements and islands.

Further, charge-state detection with AFM has been utilized for investigating the electronic structure of self-assembled InAs quantum dots (QDs) grown on a 20 nm InP tunnel barrier.^78^ This approach relies on the alternating charge transfer between a two-dimensional electron gas (2DEG) and a QD gated by an AFM tip (see Figure 5a). Once the voltage drop between the QD and the back electrode closely matches the Coulomb-blockade threshold, the tip’s oscillation introduces a gating modulation that synchronizes tunneling events with the cantilever’s motion. Analogous to experiments on intra- and intermolecular charge transfer (Figure 4c,d), charging events are detected as distinct features in the dissipation spectra upon sweeping the sample bias (Figure 5b) and as charging rings in AFM images (Figures 5c,d). This method, which relies on the charge transfer between substrate and adsorbate being gated by the cantilever oscillation, enables precise quantification of tunneling rates, charging energies, and QD interaction energies.^78,86^ Although atomic and submolecular resolution has not yet been demonstrated with this method, it has the advantage of a high degree of adaptability, allowing the investigation of samples grown even under nonvacuum conditions.^79^ Another intriguing and complementary approach for investigating and controlling charging events by means of STM makes use of gated graphene devices (see Figure 5e).^87–92^ With such an approach the charge configurations can be reversibly switched between distinct collective charge states by adjusting the graphene Fermi level through a back-gate electrode, providing access to screening clouds around ionized adatoms (see Figure 5f),^88^ as well as electron–electron interactions in molecules (see Figure 5g).^89^ In contrast to the experiments on insulators, the gated graphene surface of these devices is conductive, and single-molecule STM spectroscopy can be performed under different gating conditions and at different charge states. This way, the electron-vibron coupling has been determined for tunneling through the same molecular orbital, but in different charge states.^89^ Such exploration of charging phenomena of gated devices can help uncover how molecular-scale charging processes impact device performance.

**Figure 5:**
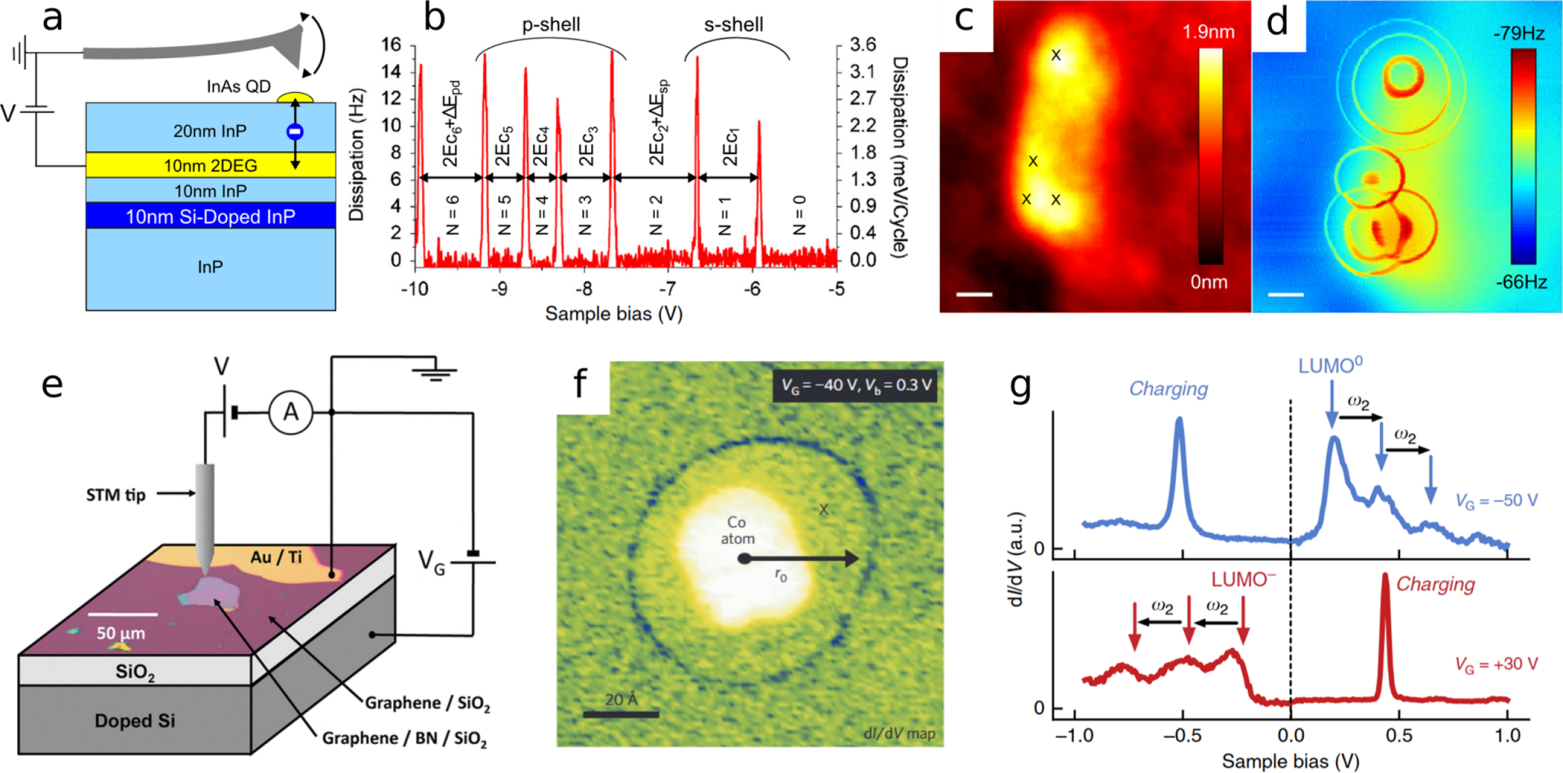
(a) Schematic of an oscillating AFM cantilever driving single-electron transfer between a two-dimensional electron gas (2DEG) and a quantum dot (QD) below the AFM tip. (b) Dissipation as a function of sample bias, revealing the quantized charging of the QD. The peaks are spaced by the Coulomb charging energy, with further contributions corresponding to energy differences (Δ*E*) between different shells. (c,d) Topography and frequency-shift AFM data, respectively, revealing QD locations and charging rings.^78^ (e) STM measurement setup for a graphene/BN device on SiO_2_. The graphene is grounded via a gold/titanium electrode, and a back-gate voltage (*V*_G_) is applied to a doped Si electrode.^87^ (f) Ionization of a Co adatom on a gated graphene device detected by a charging ring with d*I*/d*V* mapping.^88^ (g) d*I*/d*V* spectrum of a tetrafluoro-tetracyanoquinodimethane (F_4_TCNQ) molecule on gated graphene/BN reveals vibronic satellites for the molecule being at *V* = 0 V neutral (blue) and charged (red).^89^ (a–d) Reproduced figures with permission from ref 78. Copyright 2010 PNAS. (e) Reproduced with permission from ref 87. Copyright 2020 American Chemical Society. (f) Reproduced with permission from ref 88. Copyright 2011 Springer Nature. (g) Reproduced with permission from ref 89. Copyright 2016 Springer Nature.

Following an alternative strategy, charging of individual molecules has been achieved by exploiting the gating effect of charged adatoms,^93,94^ rather than the scanning tip. By manipulating native In adatoms on an InAs(111) substrate to form charged corrals, the charge state of individual free-base phthalocyanine (H_2_Pc) and copper phthalocyanine (CuPc) molecules could be controlled, leading to the realization of a prototypical single-molecule transistor.^95^ Charge-state control has also been observed on a hydrogen terminated Si(100)-(2×1) surface, where dangling bond structures could be switched between neutral, cationic, and anionic configurations. In addition, single-electron transfer between dangling bonds was detected, with implications for atomic-scale information storage and processing.^96–98^

## 6 Chemical Reactions

Charge-state control of single molecules offers the opportunity to study chemical reactions induced by individual charge injections. Combining single-electron sensitivity with submolecular spatial resolution, AFM stands out as a unique tool for monitoring chemical reactions involving different charge states of adsorbates on insulators.

Along these lines, the charge-controlled and reversible dissociation of a perylene derivative has been reported (Figure 6).^28^ The perylene derivative was functionalized with two iodine atoms, which could be dissociated by a charge injection. AFM was employed to promote the injection and removal of excess electrons as well as to visualize the resulting products and assess their charge states. By attachment of a single electron to a neutral molecule, the perylene derivative remains intact. In contrast, when charging the molecule with two excess electrons, the two iodines are split off from the molecule, yielding a radical aryne and two separated iodine anions, as revealed by AFM imaging and spectroscopy (Figure 6a,b). Notably, the chemical reaction is reversible, leading to the reattachment of iodines to the aryne by reverting the sample bias (Figure 6b–d). The high efficiency of the fragmentation process is attributed to the breaking of the halogen-carbon bonds as a result of Coulomb repulsion after the attachment of two excess electrons. It results in a neutral diradical molecule and two iodine anions on the surface. Furthermore, the halogen-carbon bonds could be reformed, when removing two electrons from the diradical molecule at negative sample bias of approximately −0.8 V (Figure 6d).^28^ The reformation is assumed to result from Coulomb attraction between the positively charged molecule and the iodine anions, resulting in the reformation of two I–C bonds and restoring the neutral initial compound. This study exemplifies how chemical reactions can be steered by the controlled injection and removal of individual electrons. Charge-driven reactions in an experimental setting in which charges can be deliberately injected into a molecule, but not escape to the substrate, result in the situation in which chemical reactions can be literally steered by one (or few) elementary charges, rendering them extremely efficient in terms of both energy and yield. In other words, the electron yield in these experiments is on the order of unity.

**Figure 6:**
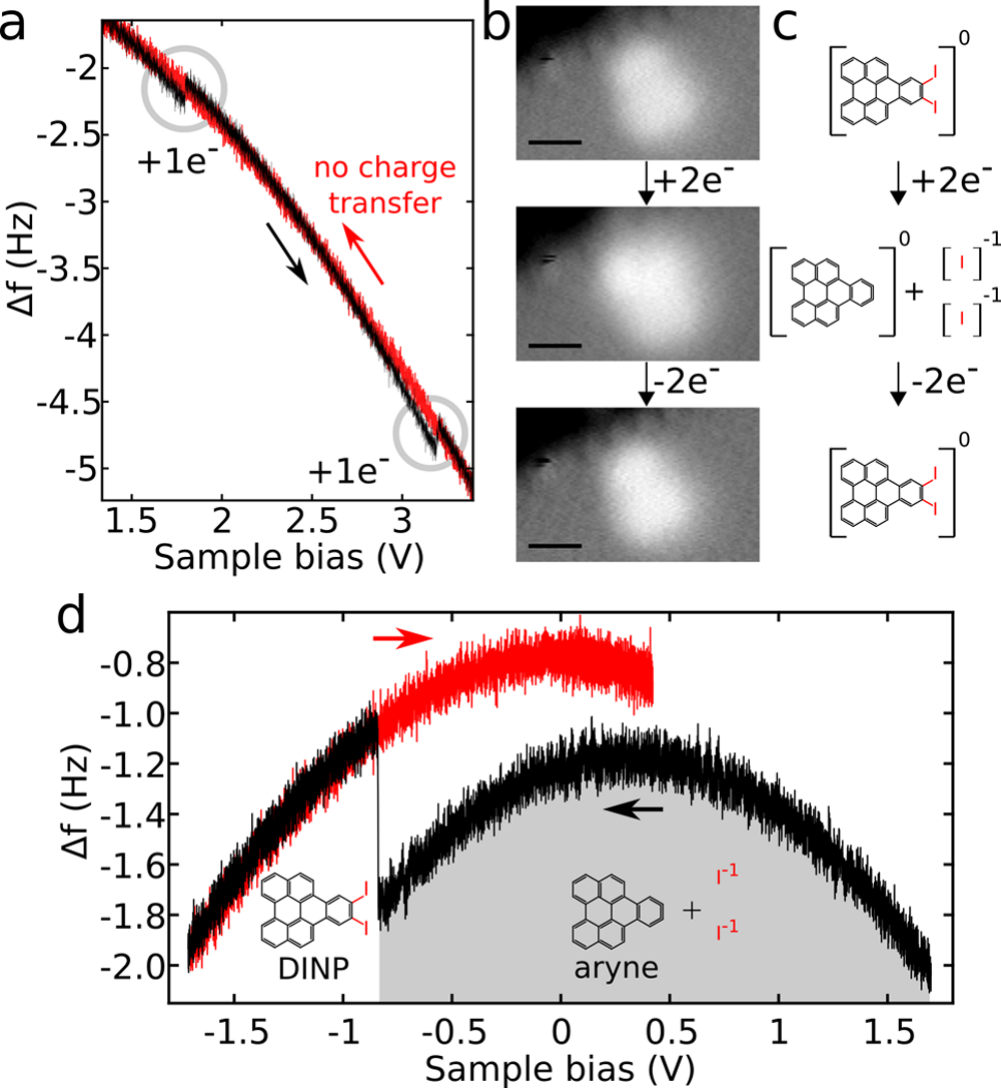
Charge-state control to break and form bonds. (a) Δ*f*(*V*) spectrum for the double negative charging of diiodo-naphthoperylene (DINP). Circles indicate charging events. (b) Sequence of constant-Δ*f* images of DINP, neutral, and prior dissociation (top panel); product after the attachment of two electrons (middle panel); restored neutral DINP after the detachment of two electrons from the dissociated system (bottom panel). (c) Reaction pathway of DINP for the double reduction of a neutral molecule and double oxidation of the dissociated system. (d) Δ*f*(*V*) spectrum of the reformation of the aryne and iodines into DINP. (a,d) Arrows indicate the sample bias-sweep direction, the first sweep in black, the successive backward sweep in red.^28^ (a–d) Reproduced figures with permission from ref 28. Copyright 2019 American Physical Society.

A similar approach, based on attaching single charges and KPFS measurements was employed to reversibly dissociate and form molecular oxygen on a semiconducting TiO_2_(110) substrate.^99^ By injecting two more electrons to already dianionic molecular oxygen [O_2_]^2–^, the system transformed into two dianionic oxygen atoms, i.e., two O^2–^. When two electrons were removed from the two neighboring dianionic oxygen atoms, the resulting product was the re-established doubly negative oxygen molecule [O_2_]^2–^.

## 7 Structural Properties

Redox processes influence the chemical properties of organic molecules, impacting aspects, such as conformation, reactivity, and aromaticity. As outlined in section 4, the combination of bond-resolved AFM imaging with charge-state control can provide detailed insights into structural changes occurring at the molecular level in response to different charge states.^24^

Bond-resolved AFM imaging in different charge states was demonstrated for a series of molecules with delocalized electron systems. The prototypical electron acceptor tetracyanoquinodimethane^100^ (T) could be stabilized and imaged in three distinct oxidation states: neutral (T^0^), anionic (T^–1^), and dianionic (T^–2^). The comparison of neutral and negatively charged species exhibits a drastic structural change: whereas the AFM image of T^0^ indicates an upstanding adsorption conformation (Figure 7a), the AFM images of T^–1^ and T^–2^ show the central carbon ring adsorbed parallel to the surface (Figure 7a). Notably, intramolecular structural changes can be detected between the two negatively charged species: The observed bond-length alternation in the central ring of T^–1^ suggested a partial quinoid character, whereas the dianion T^–2^ exhibits a homogeneous contrast on the central ring, implying a benzenoid character.

**Figure 7:**
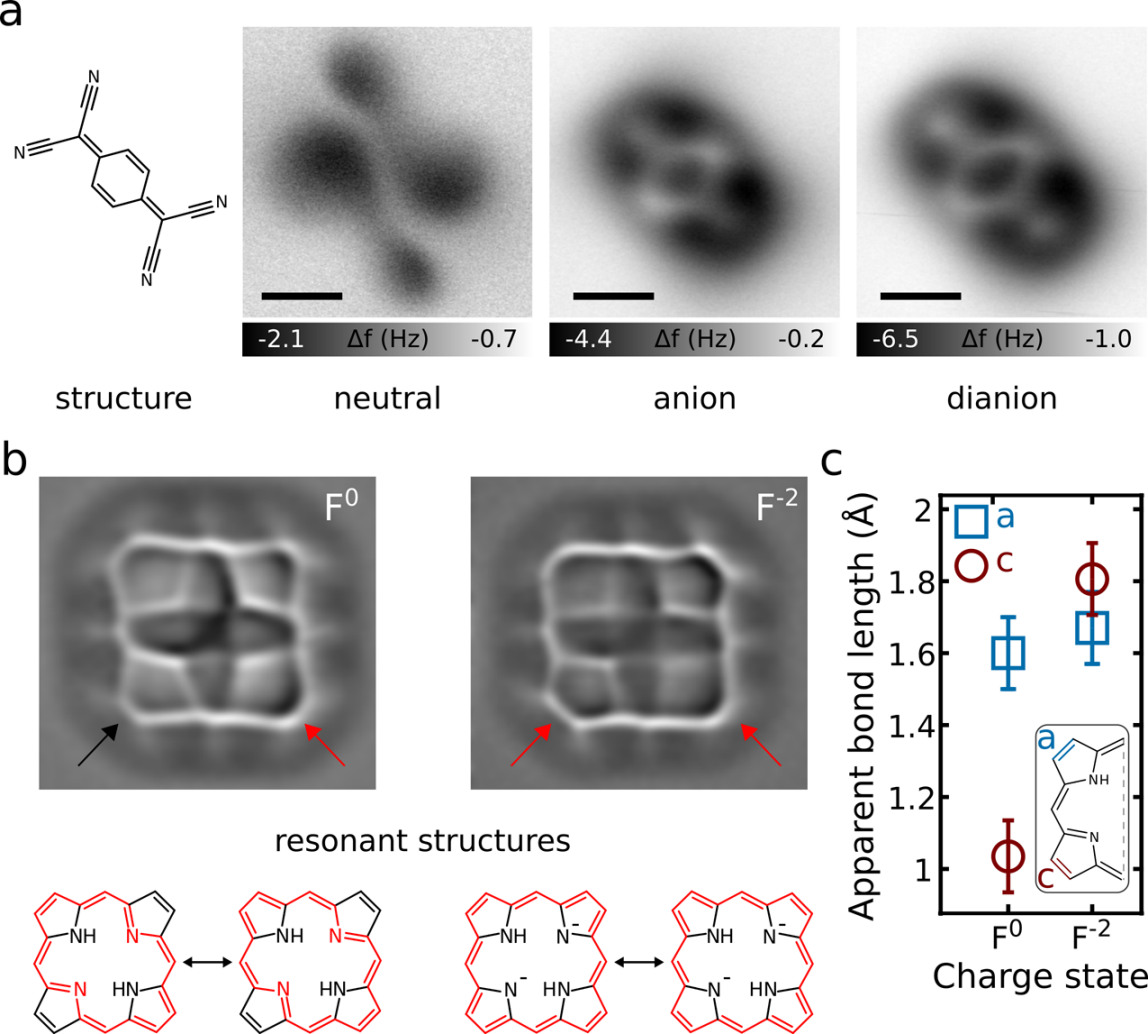
(a) Chemical structure of tetracyanoquinodimethane (T) and constant-height AFM images of the neutral, anionic, and dianionic T. Scale bars represent 5 Å. (b) Laplace-filtered constant-height AFM images of neutral and dianionic porphine (F), and below resonance structures of neutral and dianionic F. The positions of the inner hydrogens in the AFM images correspond to the displayed resonance structures. The expected annulene-type conjugation pathways are indicated in red. Note the relatively large bond order and short apparent length in the AFM image of F^0^ of the bond, which is indicated by the red arrow. This bond is not part of the conjugation pathway and is a formal double bond in both resonance structures in the neutral charge state. Its contrast changes for the dianion in which the conjugation pathway extends over this bond.^24^ (c) Measured apparent bond lengths of the peripheral C–C bonds of the pyrrole (cyan) and azafulvene (red) rings, indicated in the inset, as a function of charge state. Shown measurements on multilayer NaCl films (a–c). Adapted with permission from ref 24 Copyright 2019 AAAS.

The aromaticity and conjugation pathway of porphyrins in different oxidation states have been subjects of interest and debate,^101^ and could be investigated by bond-resolved AFM. Comparison of AFM images of porphine (F), acquired in the neutral (F^0^) and dianionic (F^–2^) charge state reveals variations in apparent bond lengths within the macrocycle, suggesting changes in aromaticity and conjugation pathway (Figure 7b). The most significant changes are observed for the peripheral C–C bonds of the pyrrole and azafulvene rings (Figure 7c). The apparent bond lengths are in line with a change of the conjugation pathway, indicated in red in the resonance structures in Figure 7b. This is most evident in the increased apparent length of the peripheral bond of the azafulvene ring in the dianion compared to that in the neutral molecule (see red arrows in Figure 7b). Note that this effect can be rationalized by this bond being a double bond in both resonance structures of the neutral molecule, because these bonds are not part of the conjugation pathway in the neutral molecule, in contrast to the dianion. In addition, bond-order analysis of F^–2^, shows increased bond-length alternation in the methine bridges, indicating reduced aromaticity compared to the neutral molecule. Moreover, it suggests that in F^–2^ one of its resonance structures (left resonance structure of F^–2^ in Figure 7b) contributes more than the other resonance structure.

## 8 Electronic Properties

For molecules adsorbed on ultrathin insulating films, STM has proven as a very powerful technique to study the electronic properties of the adsorbates. Examples range from orbital-density imaging,^54,61,102–106^ vibronic spectroscopy,^107–109^ to single-molecule luminescence^5,6,110^ to name just a few. As discussed above, however, the tunnel coupling between molecule and sample required in conventional STM experiments renders nonequilibrium charge states to be short-lived in most cases.^66,111^

To enlarge the toolbox for probing molecules on surfaces, one would ideally like to combine the basic concept of STM with the possibility of AFM to be operated on nonconducting substrates, including the possibility of charge-state control. As discussed above, the sensitivity of AFM allows tracking individual elementary charge transfers between tip and adsorbate, while the sample bias can be used to steer such charge transfer.^26,48^ As the detected process in such experiments is related to the tunneling of electrons between tip and sample, this approach is closely related to STM although the detection proceeds by means of AFM.^26,48^

### 8.1 Reorganization Energy

Electron transfer is accompanied by structural relaxations and polarization, which in turn lead to a finite reorganization energy, stabilizing the charge state. Reorganization energies^112^ critically influence electron-transfer rates and are conventionally measured in electrochemistry, as well as with optical and photoemission spectroscopies.

AFM can be used to determine reorganization energies of individual molecules adsorbed on insulating films as shown for a naphthalocyanine molecule (NPc).^38^ The reorganization energy can be extracted from a pair of charge-state transitions in opposite directions, for example, the neutral-to-positive charge-state transition (oxidation of the neutral molecule, ox^0^) and the positive-to-neutral charge-state transition (reduction of the cation, red^+^). Since the electron-transfer processes occur typically much faster than the structural polarization, we describe them as vertical (Franck–Condon) transitions, occurring at the relaxed geometry of the respective initial charge state, as depicted in Figure 8a. After each of the two transitions, relaxations occur, lowering the energy by relaxation energies λ_*+*_ and λ_0_, respectively.

**Figure 8:**
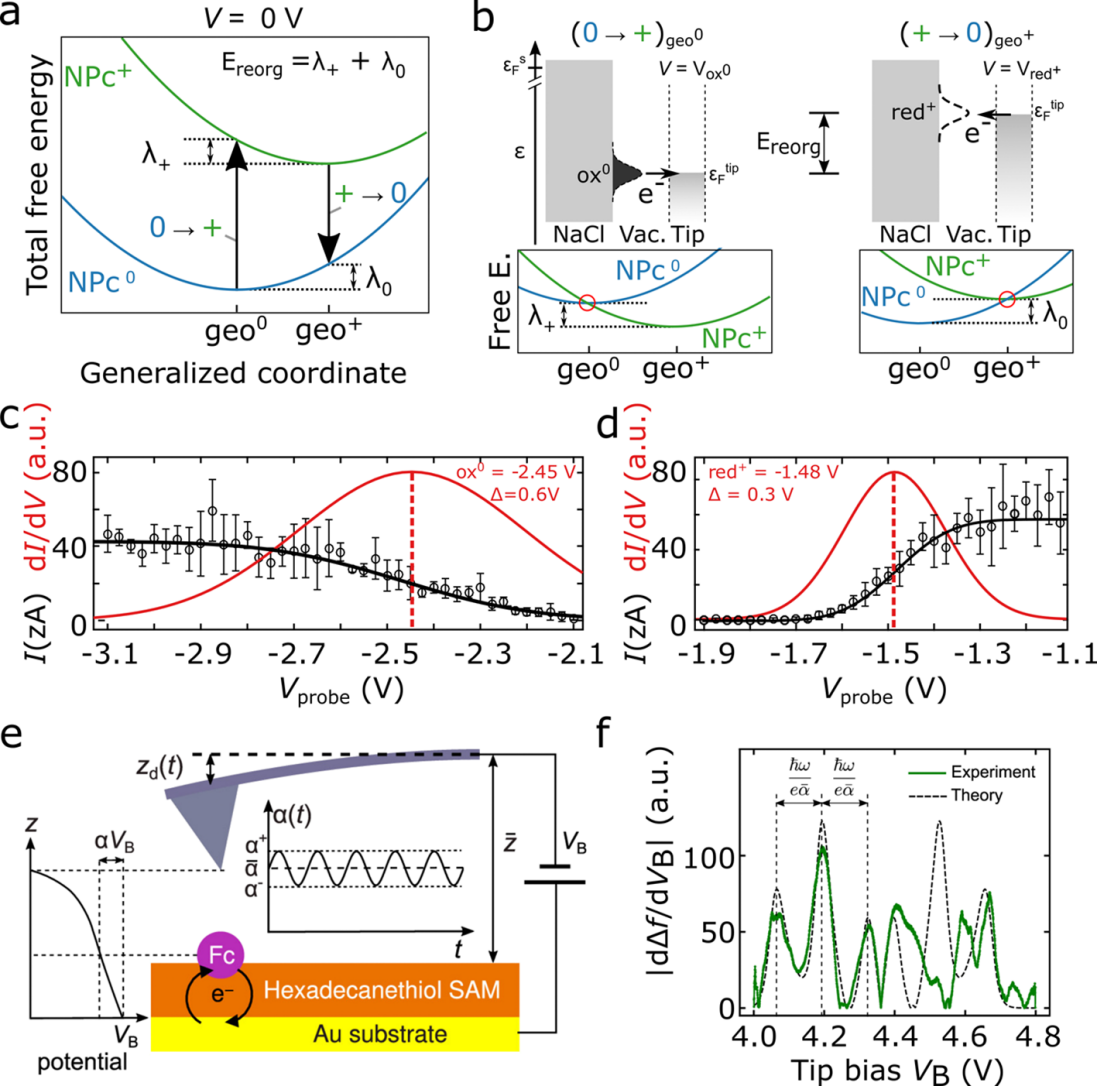
(a) Schematic of the total free-energy curve for a neutral (NPc^0^) and positively charged (NPc^+^) molecule with respect to the Fermi level of the tip. (b) Single-electron energy ε diagrams that correspond to the voltages of electron detachment from NPc^0^ to the tip (ox^0^, left) and electron reattachment from the tip to NPc^+^ (red^+^, right). The difference in electron energies corresponds to the reorganization energy. Below, the corresponding schematics for the total free energy at the respective voltage are depicted. (c,d) Extracted tunneling current *I* based on single-electron transfers and statistical analysis for a molecule adsorbed on a multilayer NaCl film. The plots also display the fitted Gauss error function (black line) and its derivative (red line). (c) Electron detachment from a neutral molecule. (d) Electron attachment to a positively charged molecule. (e) Scheme of the experiment to probe electron–nuclear coupling, where a molecule is attached to a template-stripped gold surface. (f) Derivative of the AFM frequency-shift response with respect to the tipbias *V*_B_. (a–d) Adapted with permission from ref 38. Copyright 2018 Springer Nature. (e,f) Reproduced (adapted) with permission from ref 113. Copyright 2019 American Chemical Society.

Based on charge-state control and detection as explained above, such pairs of charge-state transitions in opposite directions are accessible to experiments (Figure 8b). Upon repeatedly driving and detecting electron tunneling back and forth at different sample voltages, the transition rates as a function of voltage can be determined. Because of the stochastic nature of an individual tunneling event, such experiments are repeated many times and statistically analyzed to quantify the energies. The extracted transition rates can be converted to an equivalent of a tunneling current, shown in Figure 8c,d. By means of this method, for NPc on thick NaCl films, the reorganization energy was extracted to (0.8 ± 0.2) eV. The large ionic polarizability of NaCl is responsible for the largest fraction of the reorganization energy, whereas intramolecular relaxations contribute less.

### 8.2 Probing Electron–Nuclear Coupling

Electron-transfer processes are often accompanied by a change of the vibrational excitation, with the transition from one vibrational energy level to another being dictated by the extent of overlap between the two vibrational wave functions, as described by the Franck–Condon principle. Despite its significance, experimental access to electron–nuclear coupling is challenging given that the Franck–Condon factors are subject to thermal averaging at room temperature. Additionally, the substantial influence of the solvent on the overall relaxation poses a potential hindrance to the observation of intramolecular vibrational relaxations. As explained above for the charge transfer between adjacent moieties or molecules, the gating by the tip together with its oscillation can lead to an alternating charge transfer that is synchronized with the cantilever oscillation.^69,71,75^ The experimental setup is schematically illustrated in Figure 8e.^78,86^ The sample system consists of individual ferrocene molecules deposited on an alkanethiol layer anchored to a gold surface. Making use of this approach, Roy-Gobeil et al.^113^ reported discrete steps in Δ*f* that signify quantized vibrational excitations, consistent with a single-electron tunneling model, revealing vibronic coupling in electron-transfer processes (Figure 8f). The latter approach provides insights complementing those obtained from STS experiments of molecules on ultrathin insulating films^114–118^ as well as on gated-graphene devices.^89^

In the examples discussed in this Review, charging events typically occur in atoms and molecules deposited on a surface. However, charging can also happen in a molecule attached to the probe tip, as is exploited in the Scanning Quantum Dot Microscopy (SQDM) technique.^119,120^ In SQDM, a molecular quantum dot is attached to the tip, and by monitoring single-electron charging events, the technique is sensitive to local electrostatic fields, enabling high-resolution 3D imaging of molecular properties at the atomic scale.^119,120^

### 8.3 Imaging Charge-State Transitions

Alternating charge transfer can not only be driven by the periodic gating from the oscillating tip but also be directly steered by the application of voltage pulses to the sample: by cyclic sample-bias pulse sequences, single electrons can be transferred repeatedly back and forth between tip and molecule and detected by means of AFM. By synchronizing such cyclic sample-bias pulse sequences with the cantilever motion, one can also benefit from the enhanced sensitivity of the AFM detection to forces that occur at resonance with the oscillating tip (see Figure 9a). This way, not every charging event is detected separately, but instead charging and discharging is repeated at the time scale of the cantilever oscillation, typically tens of thousands of times per second or faster. Because of the limited bandwidth of the AFM detection, the signal already represents an average over many pulse sequences. Whereas resolving every individual charging event might be beneficial in some experiments, the large repetition rate and the intrinsic averaging has also advantages: no statistical analysis of long switching sequences is required to deduce the tunneling rates, but the latter can be directly linked to the AFM signal measured at a bandwidth that allows taking images of the resulting signal. Although the signal is detected by means of AFM, it reflects mostly the tunneling rates, being more related to what is usually being probed by means of STM. However, with this AFM approach charge-state transitions can be probed and mapped that are not accessible with STM.

**Figure 9:**
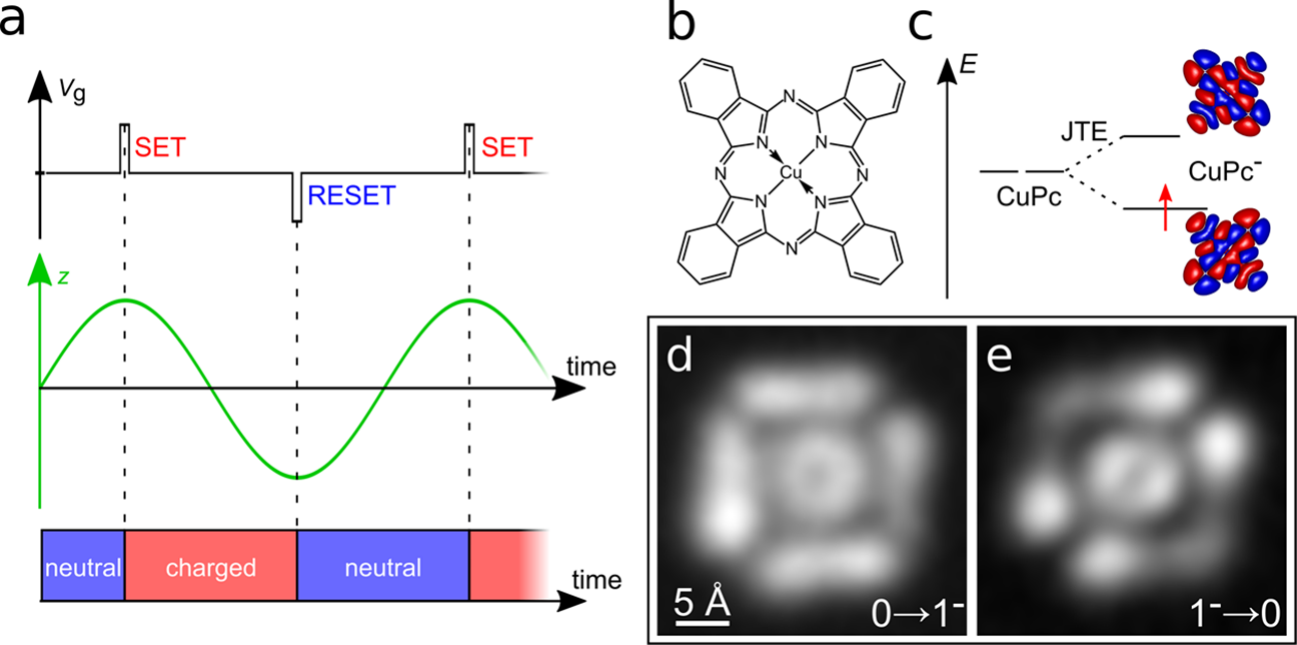
(a) Working principle of alternate-charging STM (AC-STM). Sample-bias pulses synchronized with the cantilever motion are added to a static sample voltage, driving the alternating charging (red and blue) of the molecules under the tip. The charging leads to additional electrostatic forces acting on the cantilever, adding to the background forces. (b) Molecular structure of CuPc. (c) Energy (*E*) level scheme of neutral and negatively charged molecule, showing the Jahn–Teller effect (JTE) in anionic CuPc. Calculated LUMO contours of gas-phase CuPc are shown. (d,e) Electronic transitions: 0→1^–^ (d) and 1^–^→0 (e). (a–e) Adapted with permission from ref 27. Copyright 2019 Springer Nature.

This technique, dubbed single-electron alternate-charging scanning tunnelling microscopy (AC-STM),^27^ was used to spatially map out the tunneling rates for selected charge-state transitions. Neglecting correlation effects, the spatial maps of charge attachment or detachment resemble orbital densities. Put simply, the neutral-to-cation transition (0→1^+^) is associated with tunneling out of the highest occupied molecular orbital (HOMO) and therefore resembles the HOMO density, whereas the neutral-to-anion transition (0→1^–^) resembles the lowest unoccupied molecular orbital (LUMO) density.^121^

The signal is related to repeated cycles of charging (e.g., 0→1^–^) and discharging (e.g., 1^–^→0) of a molecule.^27^ Hence, in principle, it is inevitably related to the rates of both processes. However, a trick can be used to make the signal mostly sensitive to only one of them as follows. As the tunneling events are steered by sample bias pulses, their occurrence, with respect to the cantilever oscillation cycle, can be externally controlled. By driving one of the tunneling processes at the closest and the other at the furthest turnaround point of the cantilever oscillation, their relative tunneling probabilities can be made different by (several) orders of magnitude (see Figure 9a). The combined rate of a full cycle of the two processes will then be strongly dominated by the less likely process of the two. This way, the technique can be made selective for either one of the two charge-state transitions. This selectivity was demonstrated for an individual copper­(II) phthalocyanine (CuPc) molecule making use of the Jahn–Teller effect (JTE) (see Figure 9b,c). The latter affects the molecule’s symmetry, being different for the neutral and the anionic charge state. This change of the molecule’s symmetry is directly seen in the corresponding AC-STM images (see Figure 9d,e). The possibility to spatially map transitions rates of select charge-state transitions was also used to visualize wave function localization^27,122^ as well as changes in the adsorption geometry upon charging.^123^

## 9 Electronic Excited States

The methods and experiments presented in sections 5–8 addressed the study of different charge states, but for each charge state, the molecule was in its respective electronic ground state. We will now focus on molecules in electronic excited states.

Building on the approach described in section 8.1 for the determination of reorganization energies,^38^ excited states can be created and probed spectroscopically by AFM (Figure 10a).^29,73^ After the molecule is initialized in its cationic ground state, excited states of the neutral molecule can be accessed by tunneling an electron into the molecule at higher energy. For example, instead of filling the former HOMO, an electron is tunneled into the LUMO (see Figure 10a). This is possible by applying a larger sample bias than the one required for filling the HOMO. To suppress the latter process, the tip-molecule distance is increased, reducing the overall tunneling rates. This way, the transitions to the first and second excited states of the molecule could be detected and characterized (see Figure 10b), being assigned to excited triplet *T*_1_ and excited singlet state *S*_1_, respectively. The excitation energies for NPc on NaCl films were quantified to be 0.7 eV for *T*_1_ and 1.25 eV for *S*_1_.

**Figure 10:**
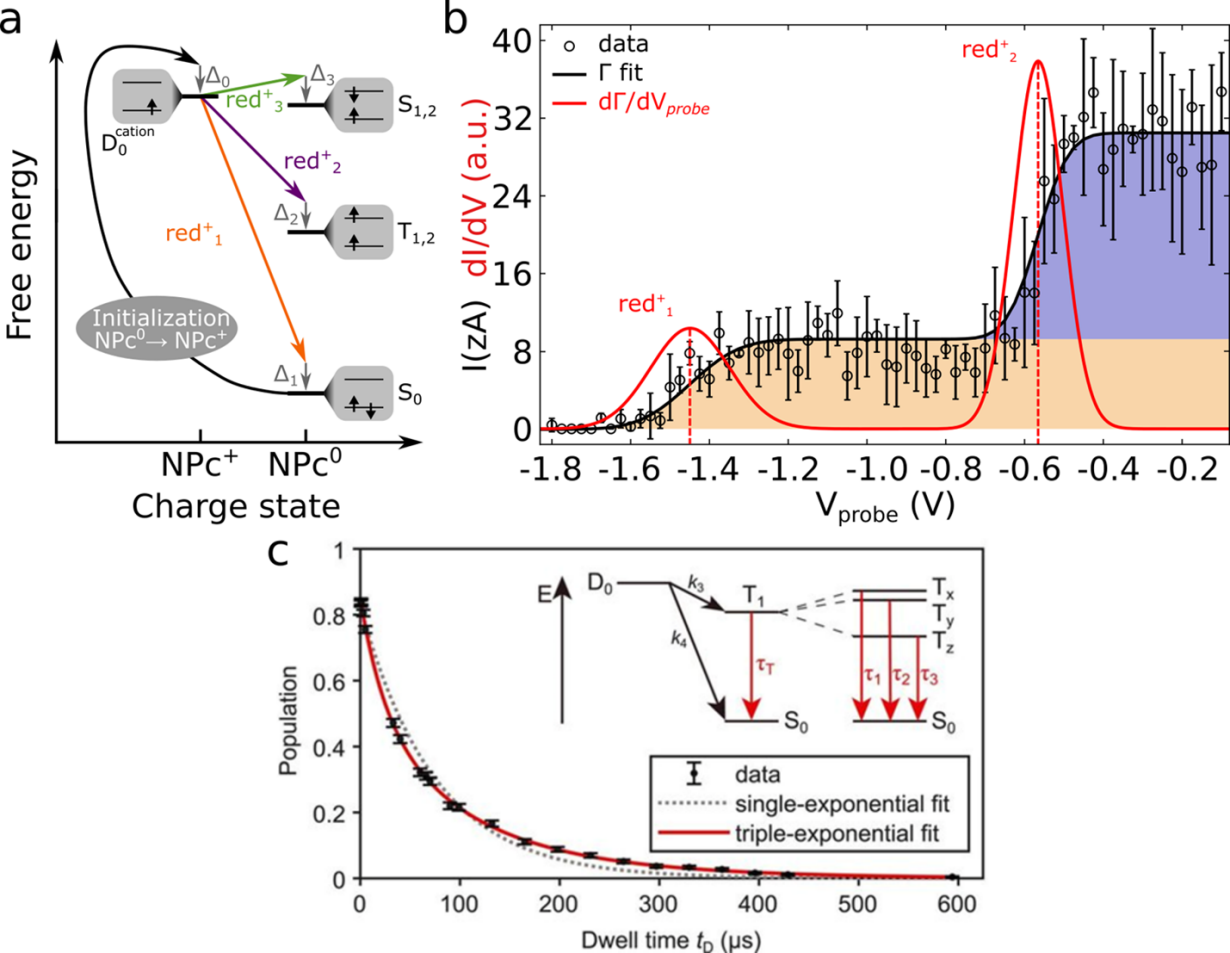
Probing electronic excited states through charge-state control. (a) Many-body electrons associated with different charge-state transitions. An increase (decrease) of sample bias shifts the cationic states upward (downward) with respect to the states of the neutral molecule. Controlled initial cation D_0_^cation^ formation is highlighted with a curved black arrow. The relaxation energies are labeled Δ_0_ (electron detachment from molecule) and Δ_1_, Δ_2_, and Δ_3_ (electron attachment to molecule). S_0_, T_1,2_, and S_1,2_ represent the ground state, energetically lowest triplet excited states, and lowest energy singlet excited states, respectively. T_1_ and T_2_ as well as S_1_ and S_2_ are quasi-degenerate because of the small energy difference between LUMO and LUMO+1 in NPc. Starting from D_0_^cation^, red^+^_1_ is the transition to S_0_, red^+^_2_ is the transition to T_1,2_, and red^+^_3_ is the transition to S_1,2_. (b) Analysis of the attachment of an electron to a cationic molecule as a function of probing sample bias, *V*_probe_. Extracted tunneling current *I* based on single-electron transfers.^29^ (c) Triplet decay of a pentacene molecule.^9^ The population as a function of dwell time *t*_D_ is extracted from repeated cycles of single-electron-transfer processes. A triple-exponential fit is used to determine the three triplet-state lifetimes involved in the transition to the ground-state S_0_. All data shown was obtained on molecules on multilayer NaCl substrates. (a,b) Reproduced figures with permission from ref 29. Copyright 2021 by the American Physical Society. (c) Adapted from ref 9. Reproduced with permission from AAAS.

Recently, the probing of excited states has been extended toward measuring their lifetime. The use of thick insulating films cannot stop electronic excited states to decay into the electronic ground state, however, it reduces exciton quenching caused by a metal surface.^124^ Measuring the lifetime of an electronic excited state requires distinguishing it from the ground state. However, ground and excited states having the same net charge are usually not expected to give rise to appreciably different AFM signals. To enable detection, ground and excited states can be projected onto different charge states,^125^ respectively, enabling electrostatic-based AFM detection. By appropriate electronic level alignment using the sample bias, only the *T*_1_ state (but not the *S*_0_ state) will transit from the neutral to the cationic state by tunneling an electron to the tip.

To measure the lifetimes, experiments have been complemented by an electronic pump–probe scheme, as previously introduced for conventional STM.^126^ Combining the all-electronic pump–probe scheme with the AFM-based access to excited states, the triplet lifetime of an individual pentacene molecule on an insulating surface has been measured,^9^ and the effect of oxygen molecules, quenching the triplet lifetime has been investigated. A pentacene molecule was first prepared in the cationic state (*D*_0_). Then, a second pulse prepared the molecule in the *T*_1_ excited state with a certain probability. During a period of controlled dwell time *t*_D_, the molecule is allowed to relax to its ground state, after which the state of the molecule is probed by mapping the ground and excited states onto different charge states. Probing the population by repeating the experiment many times for different *t*_D_ durations yields the decay of the triplet state (see Figure 10c), allowing extraction of the three different lifetimes of the three zero-field-split substates of the triplet. Moreover, the effect of molecular oxygen in triplet-state quenching was tracked at the atomic scale. Upon coadsorption of an O_2_ molecule next to pentacene, a drastic reduction of the triplet lifetime was observed, and changes in the triplet lifetime depending on the exact atomic position of O_2_ with respect to the pentacene molecule could be measured. These results show the possibility to resolve the excited-state dynamics at the atomic scale, shedding light on fundamental processes driving the photochemistry of organic materials.

Lately, such an electronic pump–probe scheme has been merged with AC-STM^27^ and the aforementioned approach to access excited states^9^ in a single experiment, by which one can map out the energies of many quantum transitions of different types, including radiative and nonradiative transitions and redox transitions, in which the charge state changes.^73^

## 10 Single-Molecule Electron Spin Resonance

Understanding and controlling decoherence in open quantum systems are crucial for quantum information processing. The implementation of electron spin resonance (ESR) in STM represents a milestone, offering access to electron spins with real-space atomic resolution.^127^ Being able to perform spin manipulation on nonconducting surfaces has the prospect of achieving long spin-coherence times, because the scattering with other electrons can be strongly reduced.

Recently, pump–probe ESR atomic force microscopy (ESR-AFM) has been introduced, enabling spin manipulation and the measurement of spin-coherence times in individual molecules.^68^ The experimental setup and pump–probe pulse scheme is similar to the triplet-lifetime experiments^9^ described in section 9. However, to enable the application of radio-frequency (RF) magnetic fields, the metallic supporting single crystal is replaced with a gold microstrip. The microstrip, also acting as a gate, is covered by a thick insulating NaCl film. Just as in the triplet-lifetime experiments, sample-bias pulse sequences drive the molecule first into the cationic and then into the neutral triplet excited state T_1_, through two consecutive tunneling events between the molecule and the conductive tip. Also in analogy to the triplet-lifetime experiments, the molecule is let to decay during a controlled dwell time *t*_D_, after which the remaining population in the triplet state is detected.^9^ In contrast to the previous experiment, during the dwell time, a RF current *I*_RF_ is passed through the microstrip to produce an RF magnetic field. Such an RF magnetic field with matching frequency can induce an ESR transition between two of the zero-field split triplet states affecting their populations, decreasing the overall lifetime of the T_1_ state.

To measure an ESR-AFM spectrum of a transition, the dwell time *t*_D_ is fixed and the triplet population is recorded as a function of frequency (*f*_RF_) of the driving field. ESR-AFM spectra reveal hyperfine interactions and characteristic features that can serve as molecular fingerprints. The spectra exhibit subnanoelectronvolt energy resolution, enabling local discrimination of molecular isotopologues. Coherent spin manipulation over tens of microseconds was demonstrated, opening research avenues for investigating the atomistic origins of decoherence and for fundamental quantum-sensing experiments.

## 11 Conclusion and Outlook

Here we reviewed the recent advances made exploiting AFM on nonconductive substrates to control and measure the charge states of individual molecules, allowing for detailed investigations of intra- and intermolecular electron transfers at the single-molecule level. The most important properties measured include electron-transfer rates, reorganization energies, redox reactions, charge-structure relationships, electronic excitation energies, charge-state lifetimes, excited-state lifetimes, spin coherence, and hyperfine interactions.

Future research could investigate more sophisticated mechanisms of intra- and intermolecular charge transfer, moving beyond gate-voltage control of charge states to encompass light-driven photoexcitation processes. These studies could leverage the charge sensitivity of AFM to track the formation and dynamics of spatially separated photocharges, offering deeper insights into excitonic states at the single molecule level. Combining this development with the emerging field of scanning-probe-based single-molecule luminescence^5,7,55,110,128–130^ would enhance the exciting research directions even further.

Looking ahead, charge-state control on insulating substrates holds great potential for achieving long coherence times of spin systems by avoiding tunneling currents, presenting opportunities to explore quantum coherence phenomena in organic molecules. ESR-AFM as reviewed in the previous section already showcases long coherence times. This approach also exemplifies how mastering charge-state control and readout can open novel research avenues beyond investigating the charging process itself. In addition, the coherent spin manipulation provided by ESR-AFM may be combined with charge-state control as a means to switch on and off mutual spin interactions as a step toward implementing functionality in spin-based quantum structures.

Furthermore, advancing tip-induced chemistry on thick insulating films offers both challenges and exciting prospects for uncovering new reaction mechanisms and molecular transformations. Recently, atomic resolution AFM images have been demonstrated on fragile and biologically relevant molecules prepared by electrospray ion beam deposition.^131–133^ Applying such sophisticated preparation methods on insulating surfaces could eventually enable atomically resolved studies of charge-transfer processes, such as long-range electron transfer^134^ in biologically relevant compounds.

Regarding the choice of substrate, the large bandgap of NaCl remains highly attractive as it enables the stabilization of multiple charge states, an essential feature for probing electron-transfer processes at the single-molecule level. Moreover, NaCl can be grown with a small defect density and chosen layer thickness on many metal substrates. NaCl leads to significant reorganization energies, resulting in large energy broadening and pronounced hysteresis in charge-state transitions. On the one side, the former, i.e., broadened peaks, and associated challenges in resolving and quantifying individual states in energy, can be considered disadvantageous for some experiments. On the other side, the latter, i.e., the large charging hysteresis is key for experiments relying on the stability of multiple charge states,^38^ such as AC-STM^27^ and ESR-AFM.^68^ Therefore, NaCl surfaces are poised to continue to play a central role in the study of charge transfer at the single-molecule level. Apart from the reorganization energy, phonons are important for the rates of nonradiative transitions and spin lifetimes. For example, in the context of achieving long spin-coherence times the low phonon density was highlighted in MgO films,^135^ emphasizing the importance of material parameters beyond bandgap size. Molecular spacers with functional groups could also be further exploited to steer the formation of self-terminating dielectric films on metal substrates.^113^ Besides, crystalline ice films^136,137^ could serve as substrate for studying the impact of water on interfacial charge formation and solvation effects.

Extending the concept of on-surface synthesis^138,139^ to insulating surfaces and combining it with the experimental concepts reviewed here bares great research potential. A few examples of thermally activated on-surface synthesis on bulk and ultrathin insulators exist.^140–145^ However, thermally activated on-surface synthesis demands a high stability of the insulator to diffusion and decomposition. For this purpose, insulators other than NaCl, e.g., covalently bonded insulators, might be more suitable. For thermally activated on-surface synthesis, and beyond, the diffusion and desorption barriers are decisive^146,147^ and may also guide the material selection for the insulating films.

Introducing the concept of lightwave-driven electronics to STM by controlling the tunneling processes with laser pulses allows studying electronic and structural dynamics at combined ultrafast temporal and atomic spatial resolution.^148–162^ Future light-wave-driven AFM could bring the research described here to ultrafast temporal resolution and open new applications in quantum sensing and single-electron control in individual molecules and molecular structures. The ultrafast temporal resolution could be exploited to time-resolve chemical reactions that can be steered by charge-state control, such as bond formation and dissociation,^28,65,163,164^ as well as configurational^105^ and conformational^24,25,63,122,123^ charge-transfer induced changes.
